# Genotype, development and tissue-derived variation of cell-wall properties in the lignocellulosic energy crop *Miscanthus*

**DOI:** 10.1093/aob/mcu054

**Published:** 2014-04-15

**Authors:** Ricardo M. F. da Costa, Scott J. Lee, Gordon G. Allison, Samuel P. Hazen, Ana Winters, Maurice Bosch

**Affiliations:** 1 Institute of Biological, Environmental and Rural Sciences, Aberystwyth University, Plas Gogerddan, Aberystwyth, Ceredigion, SY23 3EB, UK; 2 Biology Department, University of Massachusetts, Amherst, MA, USA; 3 Plant Biology Graduate Program, University of Massachusetts, Amherst, MA, USA

**Keywords:** *Miscanthus*, biofuels, plant cell wall, Fourier transform mid-infrared spectroscopy, FTIR, lignin, fermentation, bioenergy, recalcitrance, lignocellulose, biomass, development, carbohydrates

## Abstract

**Background and Aims:**

Species and hybrids of the genus *Miscanthus* contain attributes that make them front-runners among current selections of dedicated bioenergy crops. A key trait for plant biomass conversion to biofuels and biomaterials is cell-wall quality; however, knowledge of cell-wall composition and biology in *Miscanthus* species is limited. This study presents data on cell-wall compositional changes as a function of development and tissue type across selected genotypes, and considers implications for the development of miscanthus as a sustainable and renewable bioenergy feedstock.

**Methods:**

Cell-wall biomass was analysed for 25 genotypes, considering different developmental stages and stem vs. leaf compositional variability, by Fourier transform mid-infrared spectroscopy and lignin determination. In addition, a *Clostridium phytofermentans* bioassay was used to assess cell-wall digestibility and conversion to ethanol.

**Key Results:**

Important cell-wall compositional differences between miscanthus stem and leaf samples were found to be predominantly associated with structural carbohydrates. Lignin content increased as plants matured and was higher in stem tissues. Although stem lignin concentration correlated inversely with ethanol production, no such correlation was observed for leaves. Leaf tissue contributed significantly to total above-ground biomass at all stages, although the extent of this contribution was genotype-dependent.

**Conclusions:**

It is hypothesized that divergent carbohydrate compositions and modifications in stem and leaf tissues are major determinants for observed differences in cell-wall quality. The findings indicate that improvement of lignocellulosic feedstocks should encompass tissue-dependent variation as it affects amenability to biological conversion. For gene–trait associations relating to cell-wall quality, the data support the separate examination of leaf and stem composition, as tissue-specific traits may be masked by considering only total above-ground biomass samples, and sample variability could be mostly due to varying tissue contributions to total biomass.

## INTRODUCTION

Plant biomass represents an abundant resource of renewable energy in the form of cell-wall polysaccharides. Dedicated energy crops as well as dual-purpose food and energy cultivars from the Panicoideae clade, which includes *Zea mays* (maize), *Miscanthus* spp. (miscanthus), *Sorghum bicolor* (sorghum), *Saccharum* spp*.* (sugarcane) and *Panicum virgatum* (switchgrass), are C_4_ grasses that generate high yields of biomass (Feltus and Vandenbrink, 2012; van der Weijde *et al*., 2013). Among these, miscanthus represents one of the most promising dedicated second-generation bioenergy crops under development (Carroll and Somerville, 2009). Native to East Asia, members of the genus *Miscanthus* are perennial, rhizomatous plants, which remobilize nutrients to the rhizome during senescence to ensure regrowth of the crop in the subsequent season (Robson *et al*., 2012). Consequently, miscanthus is typically harvested during winter or early spring when nutrients have been translocated from above-ground tissues to rhizomes, thus providing a number of environmental advantages over annuals as bioenergy crops, including lower requirements for fertilizer, reduced soil erosion and the potential for soil carbon sequestration (Clifton-Brown *et al*., 2013). Furthermore, several varieties of miscanthus give high yields in cool climates, unusual within the C_4_ grasses, making miscanthus a potentially viable and sustainable energy crop over a wide range of diverse geographical zones (Purdy *et al*., 2013). Of the several identified miscanthus species, the most commonly investigated are *Miscanthus sinensis*, *Miscanthus sacchariflorus* and the vigorous but sterile triploid hybrids between the two, of which *Miscanthus* × *giganteus* is the most widely cultivated variety (Heaton *et al*., 2008; Dwiyanti *et al*., 2013; Liu *et al*., 2013).

Most of the potential energy in lignocellulosic biomass is locked within secondary cell walls, a heterogeneous mix of predominantly cellulose, xylan and lignin polymers that interact to assemble a complex and dense matrix (McCann and Carpita, 2008; Chundawat *et al*., 2011). The relative abundances and interactions among the polymers dictate biomass recalcitrance to saccharification (i.e. amenability to deconstruction to release fermentable sugars). Therefore, one of the key traits for the processing of plant biomass to produce biofuels and biomaterials is secondary cell-wall quality (Himmel *et al*., 2007; DeMartini *et al*., 2013).

Lignin, one of the secondary cell-wall components, consists of a complex aromatic heteropolymer composed of varying percentages of three phenylpropanoid units: *p*-hydroxyphenyl (H), guaiacyl (G) and syringyl (S) (Fukushima and Dehority, 2000; Bonawitz and Chapple, 2010). The concentration of lignin, its composition and the manner in which it binds holocellulose within the cell-wall matrix is often seen as an exacerbating factor of cell-wall recalcitrance to enzymatic deconstruction (Vanholme *et al*., 2010; Hodgson *et al*., 2011; Ding *et al*., 2012). However, the extent of this effect is not always consistent in literature reports and therefore recalcitrance should not be attributed solely to the presence of lignin (Grandis *et al*., 2014). In miscanthus, for instance, contrasting influences of lignin content on enzymatic hydrolysis have been reported (Lygin *et al*., 2011; Zhang *et al*., 2012). Other factors also influence enzymatic cell-wall hydrolysis, such as cellulose crystallinity (Hall *et al*., 2010) and hemicellulosic xylan polysaccharides (DeMartini *et al*., 2013), although the impact of these compositional and architectural features will vary depending on plant species, developmental stage and tissue type.

Despite the importance of optimizing miscanthus cell-wall properties to improve its usefulness as a sustainable and economically viable bioenergy crop, there are surprisingly few reported studies on the cell-wall composition and biology of this genus (Slavov *et al*., 2013*a*) and, to our knowledge, no reports addressing cell-wall changes as a function of tissue type and development.

To investigate the chemical, structural and biological features of miscanthus biomass as a lignocellulosic feedstock, and to unveil how these characteristics vary among different genotypes, we embarked on an in-depth cell-wall analysis of 25 miscanthus genotypes from a larger replicated field trial comprising 244 genotypes. Several earlier studies on the entire field trial have focused on a diverse set of physiological and agronomical traits, including senescence (Robson *et al*., 2012), flowering time (Jensen *et al*., 2011), and canopy duration and leaf and stem morphology (Robson *et al*., 2013). In addition, cell-wall composition of the full set of genotypes was previously determined using gravimetric analytical methods in combination with near-infrared reflectance spectrophotometry (NIRS)-based calibration models (Allison *et al*., 2011). Extending the level of detail of the latter study, we used a multidimensional approach, considering different developmental stages, stem vs. leaf compositional variability, Fourier transform mid-infrared spectroscopy (FTIR) and acetyl bromide lignin determination on isolated cell-wall biomass. Furthermore, we employed a bioassay for the determination of cell-wall digestibility as a function of the ethanol yielded after fermentation with *Clostridium phytofermentans*, an anaerobic soil bacterium that can convert a wide range of cell-wall components to ethanol, without the addition of exogenous cellulases and xylanases (Warnick *et al*., 2002; Lee *et al*., 2012*b*). Finally, we consider the possible implications of our findings in terms of future research strategies aimed at developing miscanthus into a sustainable energy crop by means of broadening our understanding of cell-wall compositional features, and impacts on biorefining.

## MATERIALS AND METHODS

### Plant material

Twenty-five genotypes of *Miscanthus* were selected from a spaced field trial of 244 accessions established in 2004 near Aberystwyth, UK (52·437848°N, 4·026688°W) described by Allison *et al.* (2011). Briefly, the trial field is on a WSW 7 % sloping field, relatively exposed to southerly and westerly winds. The trial is organized into four randomized blocks, with longer block dimensions orientated perpendicularly to the main slope, and each surrounded by a dense guard perimeter of a commercially available variety of *M*. × *giganteus*. The soil is characterized by a pH ranging from 5·1 to 6·3, and consists of a stony seasonally waterlogged loam overlying shale, with the stone fraction estimated at 50 % of the soil mass in the 0–40 cm layer. Genotypes were selected to represent a wide range of compositional variability, estimated by gravimetric measurements of neutral detergent fibre, acid detergent fibre and acid detergent lignin in bulked plant tissue samples (Allison *et al*., 2011). Priority was also given to genotypes included in genome-wide association studies (Slavov *et al*., 2013*b*). For each genotype, a single tiller of length equal or greater than three-quarters of the plant's total height (excluding rhizome and inflorescence when present) was selected randomly and collected from three of the four replicate plots. Samples were collected at three time points during the 2012–2013 growing season. The time points corresponded to three developmental stages: 10 weeks after first shoot emergence, when the plants were actively growing (AG); 18 weeks after emergence, a stage when the plants had mostly ceased their growth (peak biomass, PB); and at 42 weeks after emergence, when the plants had completely senesced (senesced stage, SS). Immediately after collection, the tillers were photographed, measured and left at –20 °C overnight, before being freeze-dried. Once dry, stem and leaf tissue (including sheath) were separated and weighed, leaf contribution was recorded as percentage of total biomass dry weight, and individual tissues were ground to a particle size in the range 0·18–0·85 mm (mesh sizes 80 and 20). By the end of the growing season, 18 samples had been collected for each of the 25 selected lines (3 developmental stages × 3 biological replicates × 2 tissues).

### Cell wall biomass preparation

All compositional analyses and the *C. phytofermentans* bioassay were carried out on purified cell wall, which was prepared following a procedure adapted from Foster *et al.* (2010). For each sample, approx. 1 g of ground plant biomass was extracted sequentially as follows: with 30 mL ethanol, first for 12 h and then twice more for 30 min in a shaking incubator set at 40 °C/150 r.p.m.; three times with 20 mL chloroform/methanol (1:1 v/v), for 30 min incubation at 25 °C and 150 r.p.m.; and finally, three times with 15 mL acetone, for 30 min, at 25 °C/150 r.p.m. Between each step of the extraction, the material was collected by centrifugation at 887 *g*/10 min and the supernatants were discarded. Following the third acetone step, the samples were left to dry overnight in a fume hood. The dried, solvent-extracted biomass was then re-suspended in 15 mL of 0·1 m sodium acetate buffer (pH 5·0) and heated to 80 °C/20 min to induce starch gelatinization followed by cooling on ice. Subsequently, samples were centrifuged and supernatants were discarded, after which the resulting pellet was washed twice with 30 mL deionized water, with resuspension, centrifugation and supernatant removal being performed for each wash. Sodium azide was added at 0·0002 % (w/v) to inhibit microbial growth, and starch was removed by incubation with type-I porcine α-amylase (Sigma-Aldrich, St Louis, MO, USA; 47 units per 100 mg cell wall) in 0·1 m ammonium formate buffer (pH 6·0) at 25 °C/110 r.p.m. After 48 h, digestion was terminated by heating to 95 °C/15 min and samples were cooled on ice. The destarched cell-wall preparations were then washed three times in 30 mL deionized water and twice with 20 mL acetone, with centrifugation and supernatant removal, before being freeze-dried.

### Fourier transform mid-infrared spectroscopy

FTIR was performed on the prepared cell-wall biomass for all miscanthus samples (25 lines × 3 time points × 2 tissues × 3 plant replicates). Duplicate spectra were collected by attenuated total reﬂectance (ATR) in the range 4000–600 cm^–1^ using an Equinox 55 FTIR spectrometer (Bruker Optik, Ettlingen, Germany) equipped with a Golden Gate ATR accessory (Specac, Slough, UK). Spectra were averaged over 32 scans at a resolution of 4 cm^–1^ and corrected for background absorbance by subtraction of the spectrum of the empty ATR crystal. Absorbance spectra were converted to text files in Opus (v. 5.0; Bruker Optik), imported into MatLab (v. R2010b; MathWorks, Natick, MA, USA) and averaged. Full spectra, or fingerprint region spectra (1900–800 cm^–1^), were transformed according to the Savitzky–Golay algorithm (order: 3; window: 15 pt), to improve peak resolution, and mean centre normalized (mean 0, s.d. 1) prior to principal components analysis (PCA) using the Eigenvector PLS Toolbox (v. 7.0.3; Eigenvector Research, Wenatchee, WA, USA) to investigate the underlying relationships between the spectra.

### Lignin measurement

Acetyl bromide lignin was determined in triplicate for all of the miscanthus samples (25 lines × 3 time points × 2 tissues × 3 plant replicates) following the general procedures described by Foster *et al*. (2010) and Fukushima and Hatfield (2004), with some modifications, described as follows. Approximately 7·0 mg of the cell-wall samples was weighed into 10-mL Pyrex glass tubes fitted with polypropylene caps. For lignin solubilization, 500 μL of freshly prepared 25 % (v/v) acetyl bromide solution in glacial acetic acid was added to the samples, the tubes were capped and placed in a heating block set at 50 °C for 2 h, after which the tubes were mixed using a vortex mixer every 15 min up to a total incubation time of 3 h. Following digestion, the tubes were cooled on ice and the contents of each were diluted by the addition of 2000 μL of 2 m NaOH. A further addition of 350 μL of 0·5 m hydroxylamine hydrochloride to each tube ensured the decomposition of polybromide ions (Monties, 1989). After vortex mixing, the final volume was adjusted to 10 mL with glacial acetic acid. The tubes were recapped, mixed by inversion and centrifuged to produce a particulate-free supernatant, and 200 μL of each sample was transferred to UV-transparent 96-well plates (UV-Star; Greiner Bio-One, Gloucestershire, UK). Absorbance at 280 nm was measured with a plate reader (μQuant; Bio-Tek Instruments, Winooski, VT, USA) using KC4 software (v. 3.3; Bio-Tek). An assay control sample of a standard cell-wall preparation was included in all batches of the lignin assay as an internal standard. Additionally, negative controls containing no cell-wall material were included and their absorbance at 280 nm was set as absorbance baseline. A specific absorption coefficient (SAC) of 17·78 g^–1^ L cm^–1^ has been reported for puriﬁed HCl-dioxane lignin from miscanthus samples (Lygin *et al*., 2011) and this was used to calculate the percentages of lignin in the cell-wall biomass samples as dry weight using the following equation: ABSL% = (*A*_280_/(*SAC*×*PL*))×(*V*_R_/*W*_S_)×100 %, where ABSL% is the acetyl bromide-soluble lignin percentage content, *A*_280_ is the absorption reading at 280 nm, *PL* is the pathlength determined for the 96-well microplates with a volume of 200 μL per well used during the analysis (0·556 cm), *V*_R_ is the reaction volume (litres) and *W*_S_ is the sample weight (g). Note that the ABSL method employed also measures ester-linked hydroxycinnamic acids and it has been reported that these act as synthetic precursors and form an integral part of the lignin macromolecule (Ralph *et al*., 1994; Ralph, 2010; Tobimatsu *et al*., 2012).

### *C. phytofermentans* bioassay of biomass digestibility

Ethanol yield analysis was performed as described previously (Lee *et al*., 2012*a*, *b*). *C. phytofermentans* strain ISDg (ATCC 700394) was cultured in a defined medium, MQM5·1, prepared as follows: 2·0 g L^–1^ NaH_2_PO_4_, 10·0 g L^–1^ K_2_HPO_4_, 1·0 g L^–1^ (NH_4_)_2_SO_4_, 1·0 g L^–1^ l-cysteine hydrochloride monohydrate, 20 mL L^–1^ XT solution (5·0 g L^–1^ xanthine and 5·0 g L^–1^ thymine in 0·06 m NaOH), 10 mL L^–1^ AA1 solution (5·0 g L^–1^ of each of the following amino acids: alanine, arginine, histidine, isoleucine, leucine, methinonine, proline and valine), and 10 mL L^–1^ trace element solution (Balch *et al.*, 1979), resazurin (1 mg L^–1^), which was added as an oxidation/reduction indicator. After autoclaving, 10 mL L^–1^ CPV3 solution (20 mg L^–1^ *p*-aminobenzoic acid, 1 mg L^–1^ biotin, 30 mg L^–1^ folinic acid, 80 mg L^–1^ nicotinamide, 5 mg L^–1^ pantethine, 2 mg L^–1^ pyridoxal hydrochloride, 30 mg L^–1^ riboflavin and 10 mg L^–1^ thiamine) was added. The *C. phytofermentans* inoculum was initially grown in MQM5·1 with 3 g L^–1^ cellobiose as a carbon source using the anaerobic techniques described by Hungate (1969). Incubations were carried out in 10-mL volumes in 18 × 180-mm tubes sealed with neoprene caps.

For the biological conversion quality assay, the previously purified leaf and stem cell-wall biomass from the three replicates of the 25 miscanthus genotypes at PB developmental stage were analysed. Approximately 20-mg portions of each sample were weighed in triplicate into autoclavable 2·2-mL polypropylene 96-well plates (Axygen Scientific, Union City, CA, USA), 0·92 mL of MQM5·1 media was added, and plates were sealed and autoclaved. Subsequently, 0·01 mL of the CPV3 solution and 0·01 mL of the prepared *C. phytofermentans* inoculum was added to each well, and the samples were incubated without shaking at 37 °C/72 h. After incubation, the plates were centrifuged and a volume of 1·0 mL of each sample supernatant was collected and filtered through a 0·22-μm syringe filter unit (Millipore Corp., Billerica, MA, USA) and 5·0 μL of each sample was analysed by high-performance liquid chromatography (HPLC). The HPLC system (Waters Corp., Milford, MA, USA) was equipped with a carbohydrate analysis column (7·8 × 150 mm IC-Pak Ion Exclusion; Waters Corp.) and a refractive-index detector. The column was operated at 30 °C with 0·0025 m H_2_SO_4_ as the running buffer at a flow rate of 0·7 mL min^–1^. The retention time for ethanol (17·84 ± 0·02 min) was determined using a commercial mix (Fuel Ethanol Residual Saccharides Mix; catalogue number 48468-U; Sigma-Aldrich) containing glycerol, glucose, maltotriose, maltose monohydrate, lactic acid, acetic acid, dextrin and ethanol. Standards were analysed at the beginning, middle and end of every distinct HPLC analysis.

### Statistical analysis

All calculations for descriptive statistics, analyses of variance and Tukey's range tests were performed using the statistical software Statistica (v. 8.0; StatSoft, Tulsa, OK, USA) at a 5 % significance level. For the leaf percentage dataset, analysis of variance (ANOVA) was used to test for the effect of genotype (25 levels) and developmental stage (three levels), and correlations were determined following natural logarithm transformation of the data, which showed a skewed distribution due to the exponential nature of tissue growth. With respect to lignin content and ethanol yield, the effect of tissue type (two levels) was also tested in addition to genotype and development factors. Tukey's tests were used for multiple comparisons between factor levels. Effect sizes were calculated as eta-squared statistics: η^2^ = *SS*_effect_/*SS*_total_ (Cohen, 1973; Levine and Hullett, 2002), where SS is the sum of squares.

## RESULTS

### Morphological diversity within *Miscanthus* genotypes

**Fig. 1.**
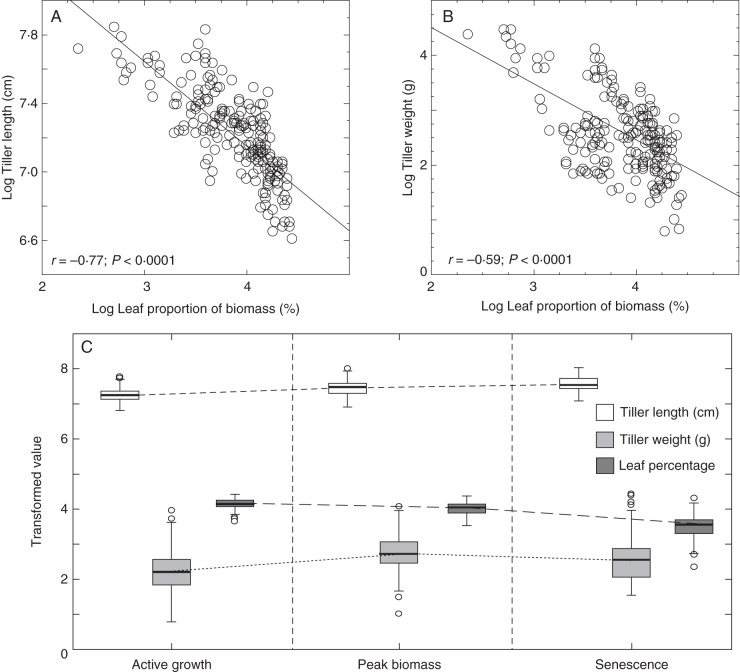
Morphological characterization of 25 miscanthus genotypes at three developmental stages. Correlation of the natural logarithms of (A) tiller length and (B) total dry weight with percentage leaf contribution to total dry weight biomass for the 25 genotypes at three developmental stages (*r*, Pearson correlation coefficient). (C) Distribution of the transformed measurements as boxplots.

**Fig. 2.**
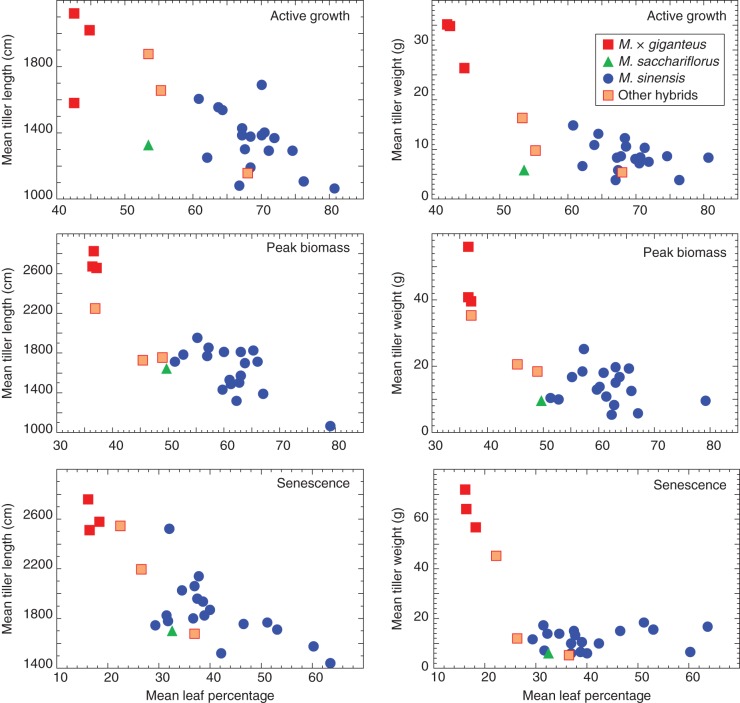
Graphical presentation of the mean leaf percentage of total biomass plotted against mean tiller length and tiller dry weight for the 25 miscanthus genotypes at three developmental stages.

The plant material used in this study included 25 miscanthus genotypes, with varying ploidy and represented two miscanthus species, *M. sinensis* and *M. sacchariflorus*, an inter-specific hybrid *M*. × *giganteus*, and other hybrids with different estimated admixture proportions of *M. sinensis* and *M. sacchariflorus* (Table 1). The mean contribution of leaf material (leaf blade and sheath) to total dry biomass among the genotypes was 63·8 % (ranging from 42·2 to 80·4 %) at the AG stage, 55·6 % (36·3–78·8 %) for PB stage and 36·1 % (15·8–63·2 %) for SS (Table 1). ANOVAs used to assess the effects of genotype, and developmental stage, on the percentage of leaf contribution to total biomass indicated that both factors were statistically significant, with large effect sizes: η^2^_genotype_ = 0·35 and η^2^_developmental stage_ = 0·51 (*P* < 0·0001). Variation in leaf percentage between the three replicates of each genotype was not significant (*P* = 0·4959), and Tukey's tests indicated that all three developmental stages were significantly different from each other. The interaction between genotype and development stage effects was not significant (*P* = 0·5858), indicating that although leaf percentage of total biomass varies throughout development, this variation is not significantly different between genotypes. The percentage of leaf contribution to total biomass was negatively correlated with tiller length (*r* = –0·77, *P* < 0·0001) and total dry weight (*r* = –0·59; *P* < 0·0001; Fig. 1A, B). Box-and-whisker plots of the distribution of leaf percentage, tiller length and tiller weight (Fig. 1C) show that the relative contribution of leaf tissue to total biomass decreased as plants matured. By contrast, tiller dry weight increased during plant growth to a maximum at peak biomass, and tiller length continuously increased until senescence, but in both cases the rate of change decreased as plants started to senesce. Mean leaf contribution to total biomass plotted against tiller length and weight, at the various developmental stages, shows that the genotypes used in this study differ in several allometric traits (Fig. 2). Throughout development hybrid genotypes ranked high in terms of tiller length and weight, but showed low leaf contributions to total biomass. This trend was predominant in *M*. × *giganteus* genotypes, whereas the other three hybrid genotypes displayed less extreme traits. The *M. sinensis* genotypes included in this study showed a broad range in leaf contribution to total biomass, whereas the *M. sacchariflorus* genotype showed a tendency to fall between the *M. sinensis* genotypes and the other hybrids.

**Table 1.** Description of the 25 miscanthus genotypes used in this study

Genotype	Species	Ploidy	Leaf percentage		
			Active growth	Peak biomass	Senesced
gig01	*M. × giganteus*	3*n*	44·53 ± 5·82	36·45 ± 0·49	16·07 ± 0·76
gig02	*M. × giganteus*	3*n*	42·30 ± 3·32	36·65 ± 3·32	17·87 ± 3·13
gig03	*M. × giganteus*	4*n*	42·23 ± 1·00	36·25 ± 0·21	15·77 ± 5·20
hyb01	55 % *M. sinensis*; 45 % M. sacchariflorus*	2*n*	55·03 ± 3·35	48·60 ± 1·56	26·33 ± 2·91
hyb02	72 % *M. sinensis*; 28 % *M. sacchariflorus*	2*n*	67·67 ± 3·36	45·10 ± 6·93	36·63 ± 3·87
hyb03	64 % *M. sinensis*; 36 % *M. sacchariflorus*	3*n*	53·20 ± 6·92	36·55 ± 0·49	22·07 ± 1·45
sac01	*M. sacchariflorus*	2*n*	53·20 ± 1·83	49·25 ± 1·91	32·07 ± 6·20
sin01	*M. sinensis*	2*n*	68·17 ± 1·10	59·40 ± 7·07	38·57 ± 2·57
sin02	*M. sinensis*	2*n*	61·80 ± 4·77	50·85 ± 6·72	31·40 ± 1·85
sin03	*M. sinensis*	2*n*	68·13 ± 22·13	60·55 ± 17·61	46·17 ± 21·27
sin04	*M. sinensis*	2*n*	74·27 ± 1·78	65·55 ± 4·88	34·23 ± 0·85
sin05	*M. sinensis*	2*n*	64·13 ± 4·43	56·85 ± 0·64	37·10 ± 3·30
sin06	*M. sinensis*	2*n*	71·57 ± 3·01	62·70 ± 0·14	52·67 ± 4·62
sin07	*M. sinensis*	2*n*	66·87 ± 2·01	56·60 ± 1·13	28·87 ± 2·51
sin08	*M. sinensis*	2*n*	69·67 ± 10·21	63·35 ± 0·21	37·40 ± 1·28
sin09	*M. sinensis*	3*n*	63·47 ± 1·01	59·70 ± 2·55	36·57 ± 1·52
sin10	*M. sinensis*	2*n*	66·97 ± 1·54	52·35 ± 2·90	36·43 ± 2·25
sin11	*M. sinensis*	2*n*	70·17 ± 0·76	60·95 ± 1·63	31·00 ± 12·91
sin12	*M. sinensis*	2*n*	76·03 ± 9·49	66·65 ± 11·81	59·87 ± 16·37
sin13	*M. sinensis*	2*n*	80·43 ± 2·50	78·75 ± 0·78	63·17 ± 11·55
sin14	*M. sinensis*	2*n*	69·73 ± 2·65	64·50 ± 0·71	50·63 ± 2·75
sin15	*M. sinensis*	2*n*	60·50 ± 1·70	54·80 ± 6·08	31·70 ± 1·60
sin16	*M. sinensis*	2*n*	66·63 ± 4·07	61·80 ± 4·81	39·47 ± 5·20
sin17	*M. sinensis*	2*n*	67·37 ± 8·71	62·45 ± 16·48	38·27 ± 9·72
sin18	*M. sinensis*	2*n*	70·90 ± 7·10	62·70 ± 0·99	41·93 ± 7·09
Overall			63·80 ± 10·14	55·57 ± 10·93	36·09 ± 12·12

Values for leaf percentage of total biomass dry weight are expressed as mean ± s.d. for the three replicated plants at the three developmental stages for each genotype.

*M. sinensis/*M. sacchariflorus* admixture proportions determined from single-nucleotide polymorphism data (Slavov *et al*., 2013*b*).

### Fourier transform mid-infrared spectroscopy

**Fig. 3.**
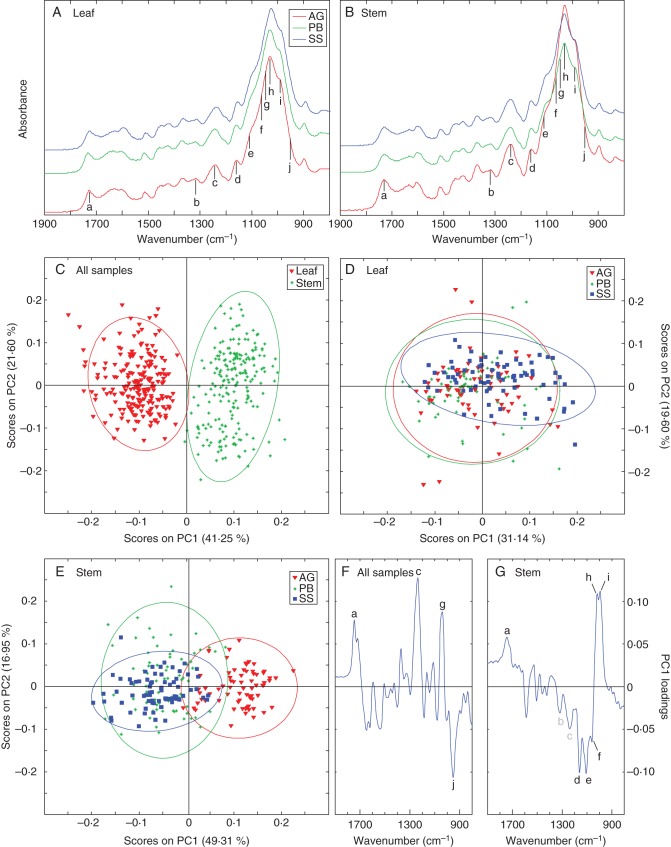
Mean FTIR spectra of (A) leaf and (B) stem samples of 25 miscanthus genotypes at three developmental stages in the range 1900–800 cm^–1^. Plot of principal component one (PC1) and principal component two (PC2) scores for (C) all samples, (D) leaf samples and (E) stem samples. PC1 loading plot for (F) all samples and (G) stem samples. Spectral bands: a, 1745 cm^–1^; b, 1325 cm^–1^; c, 1230 cm^–1^; d, 1159 cm^–1^; e, 1105 cm^–1^; f, 1060 cm^–1^; g, 1037 cm^–1^; h, 1017 cm^–1^; i, 993 cm^–1^; j, 950 cm^–1^. Abbreviations: AG, active growth; PB, peak biomass; SS, senesced stage.

FTIR spectroscopy allowed us to investigate cell-wall composition in miscanthus stem and leaf samples, as well as to identify the major compositional shifts in each of these tissues during development. Comparison of the spectra for stem and leaf samples of the 25 genotypes at each developmental stage showed differences in the relative absorbance of the individual bands. However, they were too numerous and complex for detailed visual interpretation, and PCA was employed as an exploratory approach to identify the most distinctive features of the collected spectra. Following PCA, ten spectral bands were detected as the main discriminant principal component (PC) loadings in the fingerprint region of the spectra (1900–800 cm^–1^; Fig. 3A, B). The attribution of spectral areas to their corresponding cell-wall components was made according to the literature (Supplementary Data Table S1). Bands associated with cellulose were found at 1159 cm^–1^ (d), 1061 cm^–1^ (f), 1038 cm^–1^ (g) and 993 cm^–1^ (i) (Marry *et al*., 2000; Wilson *et al*., 2000; Oh *et al*., 2005; McCann *et al*., 2007; Schulz and Baranska, 2007; Adapa *et al*., 2009; Gwon *et al*., 2010; Matos *et al*., 2013; Abidi *et al*., 2014); pectin-associated loadings at 1746 cm^–1^ (a), 1105 cm^–1^ (e), 1017 cm^–1^ (h) and 951 cm^–1^ (j) (Séné *et al*., 1994; Coimbra *et al*., 1999; Kačuráková *et al*., 2000; Wilson *et al*., 2000; McCann *et al*., 2001; Alonso-Simón *et al*., 2004); and discriminant bands associated with S lignin at 1321 cm^–1^ (b) and 1234 cm^–1^ (c) (Labbé *et al*., 2005; Gorzsás *et al*., 2011; Zhou *et al*., 2011).

Three PCA models were created, with the first one including all collected spectra (Fig. 3C). In this model, the first four PCs accounted for nearly 84 % of the variance in the spectral data set, of which PC1 captured 41·25 %. No clustering was detected concerning the various miscanthus species; however, two clear clusters were observed along PC1 comprising spectra from stem and from leaf tissue. The loadings of PC1 for this model (Fig. 3F) showed that differences in four regions (designated a, c, g and j; as described above) of the FTIR spectra were the main contributors to the differential clustering of stem and leaf samples. One prevalent positive loading, located at 1234 cm^–1^ (c), coincides with a band frequently associated with S units in core lignin. However, the other three main loadings of PC1 overlapped spectral regions typically associated with structural carbohydrate in lignocellulosic samples: positive peaks at 1746 cm^–1^ (a) and 1038 cm^–1^ (g), and a negative peak at 951 cm^–1^ (j). This indicates that PC1 is mostly correlated with portions of the spectra associated with carbohydrates. Further PCA models were created after the spectral data had been split into separate subsets comprising each tissue type: leaf (Fig. 3D) and stem (Fig. 3E). For leaf samples, the first five PCs captured slightly more than 83 % of the total variance, but no clear clusters could be discerned along any of the PC axes (shown for PC1 and PC2 in Fig. 3D). In contrast, analysis of the stem spectral data (the first four principal components accounted for almost 85 % of the variance) detected two distinctive clusters correlating to developmental stage along PC1 (Fig. 3E): one consisting of stem cell-wall samples from actively growing plants, and another of overlapping stem samples collected at peak biomass and after senescence. For this PCA model, six spectral regions featured prominently in the loadings for PC1 (Fig. 3G). These loadings, which captured 49·31 % of the variance in stem spectral data, showed that this principal component is mostly correlated with spectral regions attributed to cell-wall polysaccharide components: positively at 1746 cm^–1^ (a), 1017 cm^–1^ (h) and 993 cm^–1^ (i); and negatively at 1159 cm^–1^ (d), 1105 cm^–1^ (e) and 1061 cm^–1^ (f). In addition, it was also evident that the bands at 1321 cm^–1^ (b) and 1234 cm^–1^ (c), associated with S lignin monomers, were perceptible negative loadings, thus suggesting higher amounts of S lignin in mature stem tissues.

### Acetyl bromide lignin content

Lignin content was measured as ABSL% of cell-wall biomass dry weight (Table 2). The mean lignin content of the 25 selected genotypes increased in both tissues as plants matured, and ranged from 18·3 % in leaf tissue at AG stage to 23·7 % in stem tissue at SS stage. Additionally, lignin content was typically higher in stem samples than in leaf samples of the same genotype.

**Table 2.** Acetyl bromide lignin percentage of cell-wall biomass dry weight

Genotype	Active growth		Peak biomass		Senesced	
	Leaf	Stem	Leaf	Stem	Leaf	Stem
gig01	17·27 ± 1·38	18·72 ± 0·67	19·61 ± 0·86	22·75 ± 0·73	20·48 ± 0·84	24·45 ± 1·22
gig02	19·48 ± 0·85	19·87 ± 1·21	18·71 ± 0·15	23·39 ± 0·98	20·80 ± 0·70	24·85 ± 0·78
gig03	18·19 ± 0·40	19·74 ± 1·48	18·55 ± 0·59	24·05 ± 1·11	21·36 ± 2·88	24·44 ± 0·93
hyb01	17·08 ± 0·23	18·03 ± 1·09	19·03 ± 0·71	21·72 ± 1·80	22·18 ± 1·57	23·55 ± 0·66
hyb02	17·43 ± 0·33	19·76 ± 1·22	19·17 ± 0·15	23·15 ± 1·39	22·51 ± 0·45	23·77 ± 0·62
hyb03	17·91 ± 1·72	20·07 ± 0·89	18·52 ± 0·45	23·64 ± 0·46	20·05 ± 0·51	24·26 ± 0·62
sac01	17·38 ± 0·78	17·18 ± 1·23	19·36 ± 1·38	20·92 ± 0·13	22·02 ± 2·31	23·25 ± 1·16
sin01	19·70 ± 1·50	19·76 ± 0·69	21·47 ± 1·00	21·72 ± 0·28	23·22 ± 1·92	22·98 ± 0·04
sin02	18·63 ± 1·88	20·41 ± 1·73	20·69 ± 0·65	21·54 ± 1·30	23·59 ± 3·03	23·88 ± 0·81
sin03	18·07 ± 1·22	18·18 ± 3·11	19·24 ± 1·05	19·90 ± 2·45	23·80 ± 0·87	22·56 ± 2·19
sin04	18·68 ± 0·53	20·00 ± 0·37	19·45 ± 1·27	20·50 ± 1·30	24·14 ± 2·33	22·32 ± 0·39
sin05	18·88 ± 1·60	19·45 ± 0·81	19·31 ± 1·37	22·55 ± 0·58	23·82 ± 2·48	23·76 ± 0·87
sin06	18·95 ± 0·98	17·68 ± 1·17	20·50 ± 1·32	21·62 ± 1·16	22·22 ± 2·70	22·86 ± 0·43
sin07	18·63 ± 1·40	19·35 ± 2·22	19·85 ± 1·54	24·33 ± 1·28	24·01 ± 2·19	22·93 ± 0·75
sin08	17·95 ± 0·66	18·09 ± 2·15	19·43 ± 1·43	21·34 ± 0·35	22·81 ± 2·34	23·01 ± 1·10
sin09	18·46 ± 1·38	21·51 ± 0·65	19·24 ± 0·99	22·76 ± 0·32	24·41 ± 2·97	24·05 ± 0·96
sin10	19·73 ± 0·68	19·74 ± 1·63	20·83 ± 1·46	22·85 ± 0·70	23·11 ± 1·22	24·10 ± 0·54
sin11	18·30 ± 0·53	19·13 ± 2·61	19·33 ± 1·12	22·13 ± 0·87	22·22 ± 2·05	24·06 ± 1·27
sin12	18·03 ± 1·50	17·79 ± 2·21	18·74 ± 1·12	21·82 ± 2·67	21·68 ± 2·39	23·70 ± 1·22
sin13	17·23 ± 0·35	16·44 ± 0·14	19·41 ± 0·83	18·62 ± 3·08	21·12 ± 1·39	22·93 ± 0·53
sin14	17·28 ± 1·43	19·39 ± 2·10	19·10 ± 0·16	21·99 ± 1·66	22·12 ± 0·22	24·54 ± 0·78
sin15	19·96 ± 0·14	21·26 ± 1·56	20·77 ± 1·03	22·49 ± 1·60	23·39 ± 0·97	24·56 ± 0·94
sin16	17·59 ± 1·13	19·93 ± 0·76	18·47 ± 0·67	22·88 ± 1·66	21·89 ± 1·38	23·92 ± 0·03
sin17	17·16 ± 2·02	21·22 ± 1·13	20·85 ± 1·00	22·52 ± 0·23	22·03 ± 0·47	22·68 ± 1·16
sin18	19·20 ± 0·60	20·34 ± 1·24	20·24 ± 1·21	22·67 ± 0·76	23·68 ± 2·15	24·06 ± 0·55
Overall	18·29 ± 0·88	19·32 ± 1·29	19·59 ± 0·84	22·15 ± 1·28	22·51 ± 1·2	23·66 ± 0·71

Values are mean ± s.d. calculated for a total of 18 samples per each of the 25 miscanthus genotypes (3 plants × 3 developmental stages × 2 tissues).

The statistical significance of development, tissue and genotype effects was confirmed by ANOVA (*P* < 0·0001 for all three factors), and Tukey's tests showed distinction between AG, PB and SS, and between stem and leaf. Furthermore, the following effect sizes were determined: η^2^_developmental stage_ = 0·50, η^2^_tissue_ = 0·10 and η^2^_genotype_ = 0·06. The lignin content variation between the three plant replicates of each genotype (*P* = 0·5553), the interaction between genotype and developmental stage (*P* = 0·88558) and the interaction genotype × developmental stage × tissue (*P* = 0·2056) were all not significant. By contrast, the interactions of genotype × tissue and developmental stage × tissue were both significant (*P* < 0·0001). In light of these results, the importance of genotype and tissue on lignin content was assessed at each developmental stage individually. The resulting ANOVA showed that tissue was the only factor that had a significant effect at the developmental stages considered (*P* < 0·0001 at each of the three developmental stages). Genotype had a significant effect on ABSL% in samples collected during AG (*P* = 0·0002) and during PB (*P =*0·0052), but not in senesced samples (*P* = 0·6215). The interaction between genotype and tissue was not significant at the actively growing stage (*P* = 0·2214), but was significant during peak biomass (*P =*0·0005) and senescence (*P* = 0·0334). All these results suggest that although genotype has a significant effect on lignin content, its influence decreases over development, until it has no significant effect on lignin concentration in samples collected during senescence. This decrease in the relevance of genotype is supported by a reduction of its effect size throughout development: η^2^_AG_ = 0·30, η^2^_PB_ = 0·13 and η^2^_SS_ = 0·12.

### Lignin content and ethanol yield

**Fig. 4.**
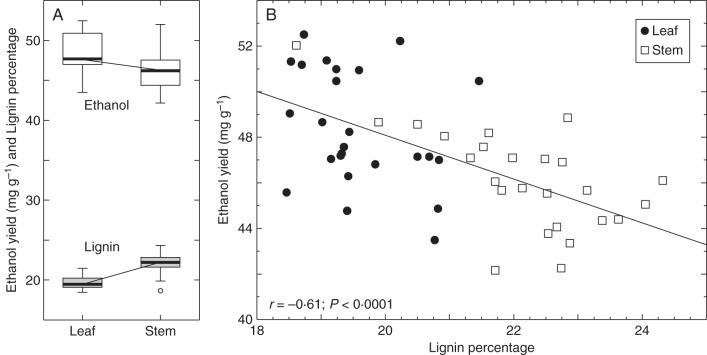
Lignin percentage of biomass dry weight and ethanol yield (mg_ethanol_ g_biomass_^–1^) for 25 miscanthus genotypes during the peak biomass developmental stage. (A) Distribution of lignin and ethanol yield measurements. (B) Least square fit of ethanol yield vs. lignin content with the associated Pearson correlation statistic (*r*).

The digestibility of stem and leaf cell-wall samples from the 25 genotypes at peak biomass was evaluated based on the ethanol concentration in the supernatant after 72 h of incubation with *C. phytofermentans*. Ethanol yields expressed as milligrams of ethanol yielded per gram of the cell-wall biomass dry weight (mg_ethanol_ g_biomass_^–1^) ranged from a maximum of 52·5 mg g^–1^ to a minimum of 42·2 mg g^–1^, in a leaf and a stem sample, respectively (Table 3). ANOVA detected that the differences in ethanol yielded by the three plant replicates of each genotype were not significant (*P* = 0·1994). By contrast, a significant difference was detected in the ethanol yields of the various genotypes (*P* < 0·0001) and between the two tissues (*P* < 0·0001), with leaf tissue typically generating higher ethanol concentrations than the stem tissue of a given genotype (Table 3), contrasting with the observed lignin contents, which were higher in stem tissues (Fig. 4A). There was a significant correlation between lignin content and ethanol yield for stem and leaf samples collected at PB, with a Pearson correlation coefficient of *r* = –0·61 (*P <*0·0001) indicating a negative association between lignin content and amenability to *C. phytofermentans*-mediated cell-wall deconstruction (Fig. 4B). Although this is a meaningful correlation, the data indicate that other factors besides lignin concentration have an exacerbating effect on recalcitrance, as supported by the individual analysis of stem and leaf data. In stem samples the interaction between ethanol yield and lignin concentration showed a coefficient of *r* = –0·65 (*P* = 0·0005). However, for PB leaf samples the interaction was not significant (*r* = –0·31, *P* = 0·1326).

**Table 3.** Supernatant ethanol concentrations as mg of ethanol yielded per g of dry cell-wall biomass after 72 h of incubation with *Clostridium phytofermentans*

Genotype	Leaf	Stem
gig01	50·90 ± 4·61	42·21 ± 7·10
gig02	51·14 ± 3·79	44·32 ± 3·63
gig03	51·30 ± 1·76	45·05 ± 1·92
hyb01	48·65 ± 6·31	42·16 ± 2·63
hyb02	47·01 ± 7·23	45·67 ± 2·69
hyb03	49·00 ± 0·82	44·36 ± 1·75
sac01	47·54 ± 2·75	48·03 ± 4·04
sin01	50·44 ± 3·30	46·04 ± 5·66
sin02	47·13 ± 2·95	47·55 ± 7·43
sin03	50·44 ± 3·91	48·62 ± 4·30
sin04	48·19 ± 6·32	48·56 ± 6·62
sin05	47·19 ± 6·14	43·73 ± 1·96
sin06	47·14 ± 1·90	48·17 ± 5·00
sin07	46·77 ± 2·12	46·09 ± 3·33
sin08	46·24 ± 2·74	47·07 ± 6·14
sin09	50·96 ± 5·25	46·88 ± 3·55
sin10	44·83 ± 4·87	48·83 ± 2·79
sin11	47·25 ± 2·08	45·73 ± 2·07
sin12	52·46 ± 3·41	45·67 ± 2·89
sin13	44·75 ± 1·33	52·02 ± 3·27
sin14	51·34 ± 2·59	47·07 ± 1·19
sin15	43·49 ± 1·60	47·04 ± 0·90
sin16	45·56 ± 3·55	43·33 ± 4·37
sin17	46·99 ± 2·66	45·51 ± 3·54
sin18	52·19 ± 5·66	44·03 ± 1·24
Overall	48·36 ± 2·53	46·15 ± 2·28

Values are mean±s.d. for six samples per each of the 25 miscanthus genotypes (3 plant replicates × 2 tissues).

## DISCUSSION

### Biomass accumulation

Most studies on cell-wall composition in energy crops use total above-ground biomass for their analysis, as this is the most relevant material for downstream applications. However, several studies focusing on the use of forage grasses and cereal straw for animal nutrition have shown that the leaf fraction is different in terms of cell-wall composition and ruminant digestibility when compared with the stem fraction (Love *et al*., 1998; McCartney *et al*., 2006).

The analysis of biomass accumulation on the 25 genotypes included in our study has shown that leaf material (blade and sheath) contributed on average to more than half of the total dry biomass during the first two harvest time points. At the senesced stage, the leaf contribution was reduced to an average of 36·1 %, mainly due to leaf abscission during senescence. In addition to the significant contribution of leaf biomass to total above-ground biomass, it is important to emphasize the variation of leaf contributions, ranging from 42·2 to 80·4 % at actively growing, 36·3 to 78·8 % at peak biomass and 15·8 to 63·2 % at senescent stages (Table 1). These varying tissue contributions can have a substantial performance and economic impact on downstream biorefining processes, as compositional differences between stem and leaf biomass will lead to tissue-specific amenability for biological conversion to ethanol (discussed below). Our data also indicate that caution is required when interpreting correlations of cell-wall phenotyping data obtained from pooled total above-ground biomass with genetic/genomic data, as part of the observed variation might actually be due to differences in the tissue contributions to total biomass.

The negative associations of leaf biomass contribution with tiller weight and length indicate that low-yield genotypes are more likely to have a higher leaf biomass percentage. However, significant differences were observed in leaf percentages across the genotypes, but not between the independent plant replicates of a given genotype. This observation suggests that the leaf to stem ratio could at least in part be a heritable genotype-specific trait, providing opportunities for the breeding of miscanthus cultivars that are simultaneously high yielders and high leaf biomass producers.

### Biomass composition

FTIR spectroscopy has become a powerful fingerprinting method to monitor modifications in plant cell-wall composition as it provides information about the main polysaccharides and lignin present in the cell wall (Kačuráková *et al*., 2000; Mouille *et al*., 2003; Derkacheva and Sukhov, 2008). Multivariate analysis of the FTIR data across the three developmental stages showed a distinct clustering of the spectra obtained from stem and leaf samples (Fig. 3C). This spectral segregation suggests significant compositional differences between stems and leaves, which is in accordance with reports in other species, such as maize, sorghum and rice (*Oryza sativa*) (Krakowsky *et al*., 2006; Murray *et al*., 2008; Jahn *et al*., 2011). These studies also showed that there is little genetic correlation of cell-wall polymer composition between leaf and stem tissues, suggesting that cell-wall composition is under separate genetic control in these tissues. Of the four spectral areas detected as prevalent loadings of PC1 (Fig. 3F), one is associated with lignin (c), while all others correlate with structural carbohydrates (a, g and j). As has been reported for sorghum (Petti *et al*., 2013), the prominence of band c in our data suggests higher amounts of S lignin in stem when compared with leaf tissues. However, given that the remaining three major PC1 loadings coincide with carbohydrate bands, it is likely that overall compositional shifts between leaf and stem cell-wall samples are more significant in their polysaccharide fractions.

Analysis of the FTIR data from different developmental stages showed that the cell-wall composition of stems from actively growing samples differs significantly from those at peak biomass or after senescence, as indicated by the discrete clusters formed during PCA (Fig. 3E). This finding most likely relates to the smaller proportion of secondary walls in actively growing stems when compared with samples at peak biomass and senesced stage. Bands associated with S lignin are noticeable negative loadings (Fig. 3G), and suggest a higher occurrence in stem samples collected during PB and SS. This is in agreement with other reports showing that more S lignin is deposited in stems when plants mature and cease to elongate, leading to a concomitant increase in the S/G ratio (Chen *et al*., 2002; Jung and Engels, 2002; Grabber *et al*., 2004). However, as above, PC1 (which is responsible for the separation between elongating and mature/senesced stem samples) is predominantly correlated with carbohydrate regions of the spectra.

Grass cell walls typically contain less pectin than their dicot counterparts (Caffall and Mohnen, 2009; Vogel, 2008). It was therefore unexpected that, in addition to cellulose, variation was detected in spectral regions attributed to pectin while there was no such variation for hemicellulose. However, the masking of bands associated with hemicellulose in the spectral region defined between 1200 and 800 cm^–1^ (Ridley *et al*., 2001) remains a possibility until further investigation reveals the precise nature of the structural polysaccharides involved. The presence of negative and positive PC1 loadings associated with pectins (a/j in Fig. 3F; and a,h/e in Fig. 3G) might indicate extensive differences in the structure and substitution of pectic polysaccharides between leaf and stem tissue and also as stems mature. This is in agreement with the fact that grasses display a marked developmental preference for accumulating differently modified pectins in specific cell types (Carpita, 1996). Furthermore, in the dicot *Linum usitatissimum* (flax), it has been shown that pectin synthesis and modification is different in stems and in leaves and that stem pectin incurs greater modifications during plant elongation (Bédouet *et al*., 2006). As for cellulose, the prominent positive band g (Fig. 3F) could indicate higher cellulose contents in stem samples. By contrast, the observed opposition of bands d, f and i in the PC1 loading plot (Fig. 3G) suggests modifications in cellulose structure as more advanced stages of maturity are reached. In effect, it has been reported that cellulose crystallinity differs between primary and secondary plant cell walls (Kataoka and Kondo, 1998; Park *et al*., 2013).

With leaf tissue samples (Fig. 3D), the compositional differences detected by FTIR were not sufficient to create PCA clusters. This possibly reflects the fact that leaf material is less changeable, and undergoes less secondary cell-wall thickening as it matures.

The ABSL values obtained for lignin content at the senesced stage were in close agreement with other values reported for several miscanthus genotypes (Lygin *et al*., 2011; Domon *et al*., 2013), although no data for actively growing and peak biomass lignin content in miscanthus are available for comparison. As expected, there was a significant developmental and tissue effect for lignin content with (1) an overall increase in lignin as the plants mature, and (2) a higher content of lignin in stem tissues than in leaf tissues. Higher stem versus leaf lignin content has been reported for a wide range of grasses, including switchgrass (Mann *et al*., 2009; Shen *et al*., 2009) and *M.* × *giganteus* (Hodgson *et al*., 2010; Le Ngoc Huyen *et al*., 2010). Our data also highlighted the limited predictive power of tissue lignin content when measured for a certain developmental stage for a specific genotype. For instance, none of the five lowest ranking genotypes for leaf lignin content at AG stage ranks among the five lowest for PB stage and only two of the highest ranking genotypes for stem lignin content at PB rank among the five highest at SS stage. The data also support the concept of distinct genetic control of cell-wall composition in leaf and stem tissue. As an example, stem tissue lignin content of the three *M*. × *giganteus* genotypes included in our study ranked among the highest five at SS, while the corresponding leaf content values ranked among the lowest five. While the overall variation in lignin content across the different genotypes remained fairly consistent for leaf tissue with increasing maturity (AG 16·9 %, PB 16·2 %, SS 21·7 %), the variation for stem lignin content is larger for AG and PB (30 and 30·7 %, respectively), but decreases at SS (11·3 %). The decrease in variation of stem lignin content may reflect a convergence in developmental variability as plants senesce, and most likely accounts for the observed absence of the genotype effect in senesced samples.

We have employed a biological assay, which uses *C. phytofer-mentans*, as a means to convert isolated cell-wall biomass to ethanol. The data obtained were subsequently correlated with the lignin concentration of the same samples, thus providing a measure of the interaction of lignin content with biomass amenability to conversion. Significant variation was observed between the two tissues and across the genotypes in terms of ethanol yields. Supernatant ethanol concentrations showed a variation of 20·6 % in leaf samples and 23·4 % in stem samples across the genotypes (Table 3). By contrast, the average ethanol yield in leaf samples was only 4·8 % higher than in stem samples, compared with the 13·1 % difference observed in the lignin concentration between the two tissues at PB. Our data suggest that the degree at which tissues are lignified does not completely account for the convertibility of lignocellulosic biomass. This is supported by the fact that drastically different coefficients were found when correlating lignin contents with ethanol yields from stem or leaf samples (*r*_stem_ = –0·65 and *r*_leaf_ = –0·31). These results indicate that lignin content has a higher relevance for the recalcitrance of stem tissue than it does for leaf tissue sampled during PB. At this stage, leaf tissue amenability to conversion may be far more influenced by other factors than it is by lignin concentration. Similar results have been reported by Le Ngoc Huyen *et al.* (2010), who found that foliar tissues show less recalcitrance than stem tissues, despite also containing appreciable amounts of lignin. Moreover, the fact that stem and leaf tissues display distinct behaviours during conversion indicates the divergent compositional arrangement of these tissues. PCA of the FTIR spectra obtained for stem and leaf samples collected at PB revealed discriminant loadings and clustering patterns along PC1 similar to those seen for the data across the three developmental stages (compare Fig. 3F with Supplementary Data Fig. S1). It is very likely that the divergent compositional features at the polysaccharide level and the lignin monomer content may be factors affecting cell-wall recalcitrance in addition to mere lignin concentration, thus making it difficult if not impossible to use the extent of tissue lignification solely as a predictor of cell-wall recalcitrance.

## CONCLUDING REMARKS

Our studies provide evidence that structural polysaccharides are main contributors to the compositional variability during stem development and between stem and leaf tissue. Hence, we hypothesize that the observed differences in recalcitrance between stem and leaf tissues are mainly attributed to divergent carbohydrate composition and cross-linking patterns between these two tissues. Variation in the relative contributions of leaf and stem tissues to total above-ground biomass, together with reports indicating that their composition is under separate genetic control, emphasize that improvement of cell-wall quality traits for the processing of miscanthus lignocellulosic biomass to biofuels and biomaterials must consider these observations. For gene–trait associations relating to cell-wall quality it is best practice to obtain leaf and stem compositional data separately as tissue-specific traits may be masked by total above-ground biomass and variability between samples could be largely due to varying tissue contributions to the total biomass.

## SUPPLEMENTARY DATA

Supplementary data are available online at www.aob.oxfordjournals.org and consist of the following. Table S1: assignment of relevant FTIR absorption bands characteristic of miscanthus cell-wall biomass. Figure S1: principal components analysis of FTIR spectra of all samples from 25 miscanthus genotypes at peak biomass stage.

Supplementary Data
