# Supplementary material for: Genotype, development and tissue-derived variation of cell-wall properties in the lignocellulosic energy crop Miscanthus
Source: Ann Bot. 2014 Apr 15;114(6):1265–77. doi: 10.1093/aob/mcu054 (PMC4195551; doi:10.1093/aob/mcu054)
Supplement: Supplementary Data [file supp_114_6_1265__index.html]

Genotype, development and tissue-derived variation of cell-wall properties in the lignocellulosic energy crop Miscanthus — Genotype, development and tissue-derived variation of cell-wall properties in the lignocellulosic energy crop Miscanthus — Supplementary Data 

# Genotype, development and tissue-derived variation of cell-wall properties in the lignocellulosic energy crop *Miscanthus*

## Supplementary Data

Supplementary Data

**Files in this Data Supplement:**

- Supplementary Data - Pdf file
