# Supplementary material for: Genotype, development and tissue-derived variation of cell-wall properties in the lignocellulosic energy crop Miscanthus
Source: Ann Bot. 2014 Apr 15;114(6):1265–77. doi: 10.1093/aob/mcu054 (PMC4195551; doi:10.1093/aob/mcu054)

SUPPLEMENTARY DATA

Table S1. Assignment of relevant FTIR absorption bands characteristic of miscanthus cell wall biomass.

Band	Wavenumber (cm^{-1})	Group	Assignment	Reference
a	1746	Polysaccharides	Ester C=O stretching associated with pectin	1745cm^{-1} (McCann *et al*., 2001; Séné *et al*., 1994; Wilson *et al*., 2000)
b	1321	Lignin	Associated with syringyl units	1320cm^{-1} (Gorzsás *et al*., 2011; Labbé *et al*., 2005)
c	1234	Lignin	C=O stretching in syringyl ring	1234cm^{-1} (Zhou *et al*., 2011)
d	1159	Polysaccharides	C-O-C stretching in cellulose	1161cm^{-1} (Abidi *et al*., 2014)<br>1159cm^{-1} (Matos *et al*., 2013)<br>1157cm^{-1} (McCann *et al*., 2007)
e	1105	Polysaccharides	Pectic polysaccharides	1104cm^{-1} (Coimbra *et al*., 1999)<br>1100cm^{-1} (Kačuráková *et al*., 2000)<br>1105cm^{-1} (McCann *et al*., 2001)
f	1061	Polysaccharides	C-O stretching and O-C-H in-plane bending vibrations in cellulose	1060cm^{-1} (Adapa *et al*., 2009; Schulz and Baranska, 2007; Wilson *et al*., 2000)
g	1038	Polysaccharides	C-O, C=C and C-C-O vibrational stretching in cellulose	1035cm^{-1} (Adapa *et al*., 2009; Schulz and Baranska, 2007; Wilson *et al*., 2000)
h	1017	Polysaccharides	Pectic polysaccharides	1014cm^{-1} (Coimbra *et al*., 1999)<br>1017cm^{-1} (Kačuráková *et al*., 2000)<br>1018cm^{-1} (McCann *et al*., 2001)
i	993	Polysaccharides	C-O stretching in cellulose	993cm^{-1} (Gwon *et al*., 2010)<br>990cm^{-1} (Marry *et al*., 2000)<br>993cm^{-1} (Oh *et al*., 2005)
j	951	Polysaccharides	Pectic polysaccharides	950cm^{-1} (Alonso-Simón *et al*., 2004)<br>952cm^{-1} (Coimbra *et al*., 1999)<br>951cm^{-1} (Kačuráková *et al*., 2000)

See main text for references.

Fig. S1. Principle component analysis of FTIR spectra of all samples from 25 miscanthus genotypes at Peak Biomass stage. Left panel: plot of principal component one (PC1) and principal component two (PC2) scores for all samples at Peak Biomass. Right panel: corresponding PC1 loading plot. Spectral bands: *a*, 1745cm^{-1}; *c*, 1230cm^{-1}; *g*, 1037 cm^{-1}; *j*, 950cm-*1*.

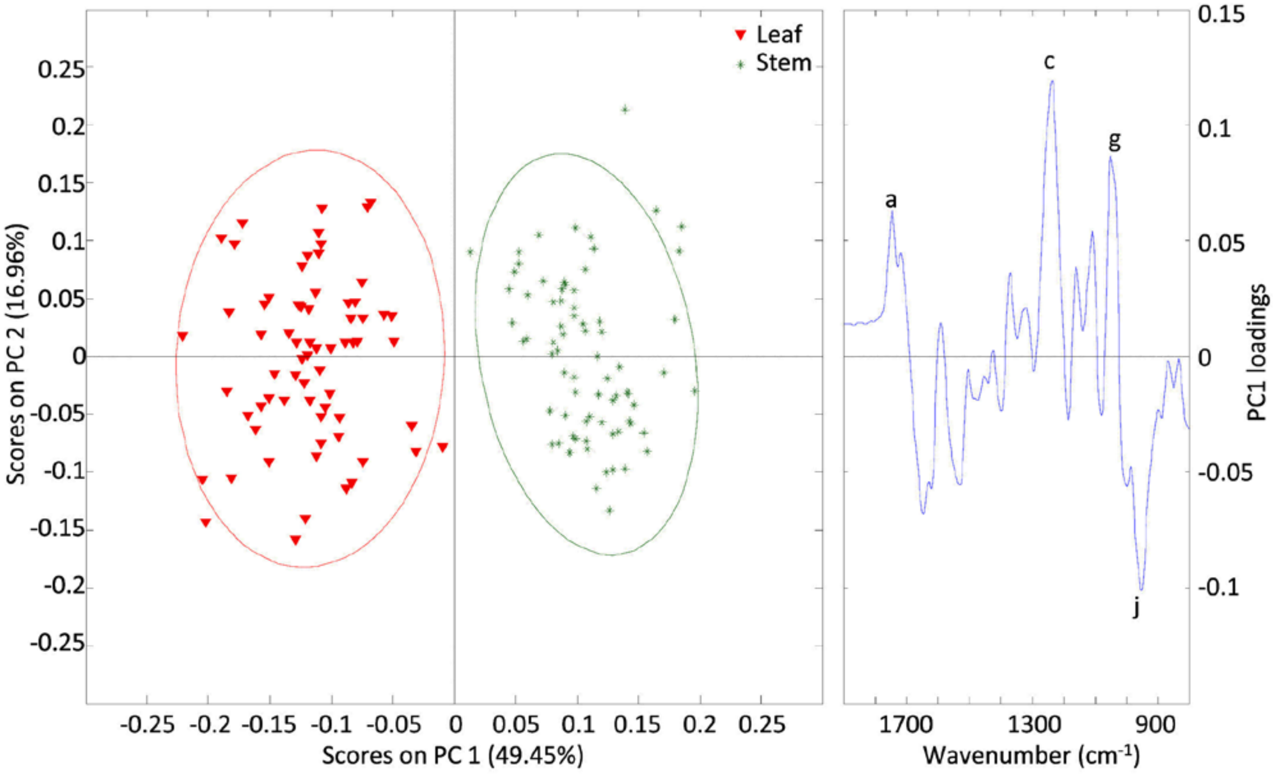

Supplement: Supplementary Data [file supp_mcu054_mcu054supp.pdf]
